# Farnesyl transferase inhibitors induce extended remissions in transgenic mice with mature B cell lymphomas

**DOI:** 10.1186/1476-4598-7-39

**Published:** 2008-05-19

**Authors:** Kenneth A Field, Soratree Charoenthongtrakul, J Michael Bishop, Yosef Refaeli

**Affiliations:** 1 Cell Biology and Biochemistry Program, Biology Department, Bucknell University, Lewisburg, PA, USA; 2 G. W. Hooper Research Foundation and Department of Microbiology and Immunology, University of California, San Francisco, CA, USA; 3 Department of Pediatrics, Program in Cell Biology, National Jewish Medical and Research Center, Denver, CO 80206, and University of Colorado Cancer Center, Aurora, CO, USA

## Abstract

**Background:**

We have used a mouse model based on overexpression of *c-Myc *in B cells genetically engineered to be self-reactive to test the hypothesis that farnesyl transferase inhibitors (FTIs) can effectively treat mature B cell lymphomas. FTIs are undergoing clinical trials to treat both lymphoid and non-lymphoid malignancies and we wished to obtain evidence to support the inclusion of B cell lymphomas in future trials.

**Results:**

We report that two FTIs, L-744,832 and SCH66336, blocked the growth of mature B cell lymphoma cells *in vitro *and *in vivo*. The FTI treatment affected the proliferation and survival of the transformed B cells to a greater extent than naïve B cells stimulated with antigen. In syngeneic mice transplanted with the transgenic lymphoma cells, L-744,832 treatment prevented the growth of the tumor cells and the morbidity associated with the resulting lymphoma progression. Tumors that arose from transplantation of the lymphoma cells regressed with as little as three days of treatment with L-744,832 or SCH66336. Treatment of these established lymphomas with L-744,832 for seven days led to long-term remission of the disease in approximately 25% of animals.

**Conclusion:**

FTI treatment can block the proliferation and survival of self-reactive transformed B cells that overexpress *Myc*. In mice transplanted with mature B cell lymphomas, we found that FTI treatment led to regression of disease. FTIs warrant further consideration as therapeutic agents for mature B cell lymphomas and other lymphoid tumors.

## Background

We have tested farnesyl transferase inhibitors (FTIs) using a mouse model of mature B cell lymphoma to determine if these drugs may be useful in treating similar lymphoid cancers. Although FTIs were originally developed to block the activation of the Ras family of oncogenes, they are also effective in blocking the growth of tumor cells that do not contain mutations at any of the *Ras *alleles [1]. By blocking the normal processing and subcellular targeting of most farnesylated proteins in the cell, FTI treatment can have many effects. This is due to the large number of farnesylated proteins present, including proteins of the Rho family that are known to mediate antigen receptor signaling in B cells. We therefore chose to test the efficacy of FTIs against our murine B cell lymphoma model, even though there is presently no evidence that activation of Ras plays a role in genesis of the tumors.

The two FTIs that we tested are L-744,832 and SCH66336 (Sarasar, lonafarnib). Developed by Merck, L-744,832 is a peptidomimetic competitive inhibitor of farnesyl transferase that blocks the binding of CAAX peptide substrates. L-744,832 has been shown to block the growth of a variety of tumor cell lines *in vitro *[2-4], nude mouse xenografts of human tumor cell lines [5], and mouse tumor models [6-11]. SCH66336 was developed by Schering Plough, completed Phase I clinical trials [12-14], and is currently in Phase II [15] and Phase III clinical trials. *In vitro*, SCH66336 has been shown to cause cell death in tumor cell lines [16-18]. Preclinical studies demonstrated that SCH66336 is orally bioavailable and could block the growth of human tumor cells in mouse xenografts [19] and of mouse tumor cells in transgenic models [17,19-21]. The efficacy of L-744,832 and SCH66336 does not appear to correlate with the expression of activated Ras protein in either human or murine tumors. Although these two FTIs have been tested in other preclinical models [22], the efficacy of this class of drugs has not been examined in clinical trials with B cell lymphoma patients.

Certain lymphoid malignancies are sensitive to FTI treatment [23], suggesting that FTIs can affect the proliferation or survival signaling pathways in lymphocytes. The growth of large cleaved cell lymphomas in transgenic mice expressing an *N-Ras *oncogene driven by the MMTV promoter can be prevented by SCH66336 treatment [8]. Transformed lymphocytes from T cell ALL patients activate cell death when treated with the FTI R115777 *in vitro *[24]. In addition to their effects on cancer cells, FTIs have also been shown to affect normal lymphocyte signaling. T cell proliferation stimulated by antigen receptor activation can be blocked by the FTIs cinnamaldehyde [25] and A-228839 [26]. The dual prenylation inhibitor, L-778,123, which blocks both farnesylation and geranylgeranylation, blocks T cell proliferation activated either by antigen receptor-stimulation or by interleukin-2 (IL-2), without affecting IL-2-mediated survival [27]. Statins, which indirectly affect farnesylation and geranylgeranylation through mevalonate biosynthesis, are also known to have immunomodulatory effects [28].

We have used a mouse model in which the overexpression of the proto-oncogene *c-Myc *creates a breach of tolerance in B cells [29]. The self-reactive B cells in these mice generate a mature B cell lymphoma that closely resembles Burkitt's lymphoma in humans [30]. The mice express three transgenes: (A) the oncogene *c-Myc *expressed from the Eμ immunoglobulin (Ig) heavy chain promoter, (B) the pre-rearranged Ig heavy and light chains specific for hen egg lysozyme (HEL) expressed from the endogenous Ig promoter, and (C) secreted HEL expressed from a metallothionine promoter. The majority of B cells in the Eμ-*Myc*/BCR^HEL^/HEL transgenic mice express only BCR^HEL ^IgM on their surface and are specific for the self-antigen, HEL. Tolerance to this self-antigen is overcome by the over-expression of *c-Myc *in these cells [29] and the resulting autoreactive B cells are hyperproliferative and form tumors in the lymph nodes and spleen. The lymphoma phenotype occurs in 100% of the Eμ-*Myc/*BCR^HEL^/HEL transgenic mice with a median latency of 8 weeks [30]. The tumors can be transplanted into unmanipulated C57BL/6 mice and the transplant recipients uniformly develop tumors after 3–4 weeks that appear identical to those in the transgenic donors. This transgenic mouse line is similar to other lymphoma models that overexpress *c-Myc *[31-34], except for context of overexpression, in autoreactive B cells. Antigen receptor activation may be similarly involved in tumorigenesis in certain human lymphoid malignancies [35].

The development of the Eμ-*Myc*/BCR^HEL^/HEL mice permits preclinical testing in this model in an effort to ascertain whether clinical trials of FTIs on mature B cell lymphomas, such as Burkitt's lymphoma, might be in order. In this report, we have demonstrated that FTIs can disrupt the growth and survival pathways in the murine tumor cells. Mice with transplanted tumors showed responses to either FTI, demonstrating effectiveness *in vivo *of this class of drug against this malignant B cell lymphoma. These preclinical results may have important implications for the treatment of B cell lymphomas in humans, particularly those whose proliferation or survival are dependent on antigen activation.

## Results

### Mouse B cell lymphoma cells are sensitive to L-744,832 *in vitro*

**Figure 1:**
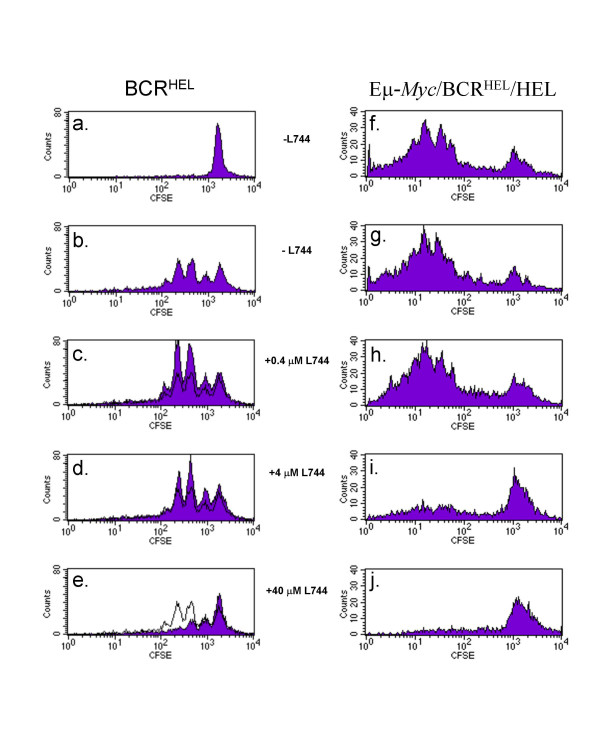
**Proliferation of mouse lymphocytes and lymphoma cells *in vitro *in the presence and absence of L-744,832**. Splenocytes were harvested from either a BCR^HEL ^transgenic mouse (panels *a*-*e*) or a moribund Eμ-*Myc*/BCR^HEL^/HEL transgenic mouse (panels *f*-*j*). The splenocytes were depleted of T cells and labeled with CFSE. Cells were cultured in RPMI (panels *a*, *f*-*j*) or RPMI with 2 μg/ml anti-IgM and 1 μg/ml anti-CD40 (panels *b*-*e*) and 0 – 40 μM L-744,832 were included in the culture media, as indicated. After three days, cellular proliferation was measured by flow cytometry. Cells that have proliferated measure less than 10^3 ^fluorescence units. For comparison, panels *c, d*, and *e *show proliferation in the absence of L-744,832 as a black line.

In order to evaluate whether the murine mature B cell lymphoma might respond to FTIs, we measured proliferation of tumor cells *in vitro*. The tumor B cells from Eμ-*Myc*/BCR^HEL^/HEL transgenic mice proliferated in the absence of exogenous stimulation (Figure 1, panel *f*). In contrast, naïve B cells from mice carrying only the BCR^HEL ^transgene did not proliferate without stimulation (Figure 1, panel *a*), but showed several rounds of cell division when stimulated with a mixture of anti-IgM and anti-CD40 (Figure 1, panel *b*). The growth of the murine tumor cells in culture provided the opportunity to test the efficacy of experimental compounds such as FTIs.

Proliferation of the mouse lymphoma cells can be blocked by the FTI, L-744,832. At relatively low concentrations of this drug (0.4 μM, Figure 1, panels *c *and *h*), there was no antiproliferative effect on either nontransformed or tumor B cells. At 4 μM L-744,832, a slight effect was seen on the nontransformed B cells (Figure 1, panel *d*), but the proliferation of the tumor cells was almost completely blocked (Figure 1, panel *i*). At a concentration of L-744,832 that was not likely to be pharmacologically relevant (40 μM, panels *e *and *j*), the proliferation of both cell types was almost completely blocked. From these data, we concluded that L-744,832 prevented the proliferation of the mouse lymphoma cells *in vitro *and that, under these conditions, it had a greater effect on the tumor cells than on the proliferation of activated, non-transformed B cells stimulated by antigen receptor and CD40 activation.

### Efficacy of L-744,832 *in vivo*

**Figure 2:**
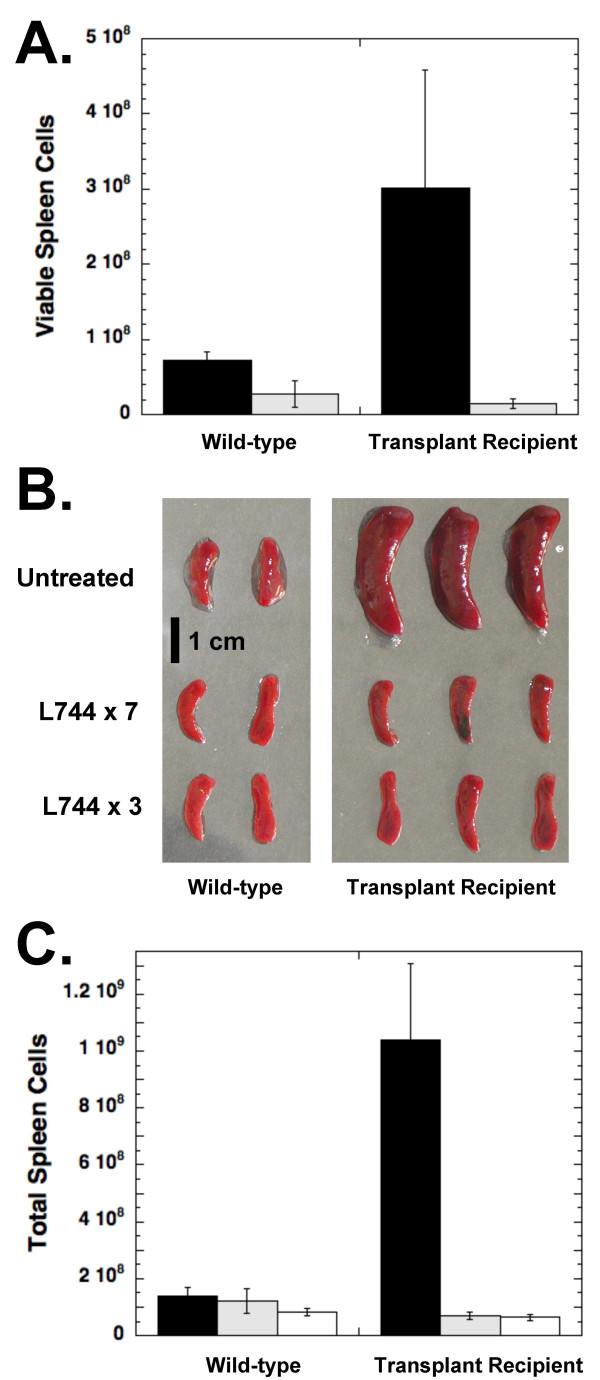
**Treatment of lymphoma-bearing mice with L-744,832**. **A**. L-744,832 treatment prevents the B cell lymphoma from becoming established. Unmanipulated C57BL/6 mice (Wild-type) or mice transplanted with 10^6 ^Eμ-*Myc/*BCR^HEL^/HEL transgenic lymphoma cells (Transplant recipient) were injected intravenously with 625 μg L-744,832 in 0.25 ml PBS daily (light bars) or left untreated (black bars). After 28 days, mice were euthanized and the spleens were dissected and viable cell counts of splenocytes resuspended in RPMI were determined using a hemocytometer. Error bars represent the standard deviation calculated for all mice in each group (n = 4). **B **and **C**. L-744,832 treatment causes rapid regression of lymphomas. In a separate experiment, mice transplanted with transgenic tumor cells were allowed to develop tumors for 20 or 24 days before daily treatment with L-744,832 as above for either 7 days (L744 × 7) or 3 days (L744 × 3), respectively. Twenty eight days following the tumor transplantation the mice were euthanized. Spleens were removed from tumor recipient mice and C57BL/6 control mice and photographed. Single cell suspensions from the spleens of mice that were left untreated (black bars), treated for the final 7 days (shaded bars), or treated for the final 3 days (clear bars) were counted using a hemocytometer. Data shown are the average of 4 mice in each group and error bars represent the standard deviation.

We next utilized our ability to transplant the murine B cell lymphoma into syngeneic mice to test the efficacy of FTIs in the mouse model. For four separate experiments, lymphoma cells were freshly isolated from the lymph nodes and spleen of an Eμ-*Myc*/BCR^HEL^/HEL transgenic mouse and 10^6 ^cells were transplanted into each of a cohort of C57BL/6 recipients. Figure 2A shows, for one of the experiments, the number of spleen cells isolated from mice that were transplanted with 10^6 ^tumor cells 28 days earlier or C57BL/6 mice that had not received tumor cells. Flow cytometry confirmed that the additional cells in the spleen were activated B cells (B220^+ ^and CD69^+^) that express BCR^HEL ^(IgM^a^) on their surface (data not shown). These observations indicate that the transplanted cells have formed lymphomas in the recipient mice.

In this experiment, four mice in each group were treated once daily with 625 μg of L-744,832 (~40 mg/kg) intravenously starting the day after tumor transplantation and four mice were left untreated. Daily administration of L-744,832 prevented the transplanted tumors from causing splenomegaly in recipient mice (Figure 2A). Treatment of the wild-type mice also resulted in a small, but significant, decrease in the number of isolated splenocytes and the numbers of viable cells isolated are similar for the two groups of FTI-treated mice.

Figures 2B and 2C show the results from a separate experiment where the recipients were not treated immediately after transplantation of the tumor. Transplant recipients either were treated for 7 days beginning 21 days after transplantation (L744 × 7), when enlarged lymph nodes were visible upon external examination, or were treated for 3 days beginning 24 days after transplantation (L744 × 3), when the mice were beginning to show debilitation due to the lymphomas. The mice were then treated with L-744,832 as above, or left untreated, for comparison. The administration of L-744,832 for either 7 or 3 days caused a large reduction in the size of the spleen (Figure 2B), as well as the lymph nodes and thymi (data not shown) and a corresponding drop in the number of cells isolated from the spleen (Figure 2C). The treated mice became more active and resumed grooming behavior within two days of the start of treatment. Similar results were observed in all four experiments using L-744,832. Together, these experiments show that L-744,832 can prevent mature B cell lymphomas from becoming established in mice and can cause established tumors to regress.

### Efficacy of SCH66336 *in vivo*

**Figure 3:**
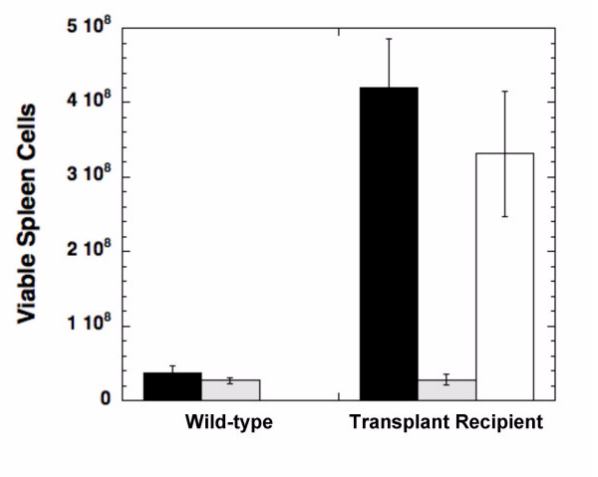
**Treatment of lymphoma-bearing mice with SCH66336**. SCH66336 treatment for 3 days causes B cell lymphomas to regress in mice. The numbers of viable, isolated splenocytes are shown from unmanipulated C57BL/6 mice (Wild-type) or mice transplanted 18 days earlier with 10^6 ^lymphoma cells from an Eμ-*Myc*/BCR^HEL^/HEL transgenic mouse (Transplant Recipient). Mice were either left untreated (black bars), treated orally for the last 3 days with 1.56 mg SCH66336 every 12 hours (shaded bars), or treated orally for the last 3 days with vehicle alone every 12 hours (clear bar). Each group contains 4 mice and error bars represent the standard deviation for each group.

We wanted to see if a distinct FTI, SCH66336, could have similar effects on the B cell lymphoma model. In three separate experiments, which all yielded similar results, we transplanted 10^6 ^transgenic tumor cells into a cohort of C57BL/6 recipients. Approximately 2 weeks after transplantation, some of the mice were treated by oral gavage with 1.56 mg SCH66336 (~100 mg/kg) or with vehicle every 12 hours. After 3 days of treatment, the mice were euthanized and splenocytes were isolated for analysis. Mice that were not treated with FTI showed an average 10-fold increase in the number of splenocytes isolated, indicating growth of the lymphoma from the cells transplanted 18 days earlier (Figure 3). Mice treated for 3 days with SCH66336 showed a large decrease in the number of isolated splenocytes, resulting in numbers equal to unmanipulated C57BL/6 mice. Tumor recipient mice that were treated with a vehicle control instead of SCH66336 actually showed a slight decrease in the number of splenocytes, although this was not consistently seen in other experiments. During SCH66336 treatment both tumor recipient and untransplanted mice were adversely affected by the drug, as demonstrated by weight loss and lack of activity. Because of these effects, we were unable to extend the treatment at this dosage past 3 days.

### Flow cytometry analysis of lymphocytes from FTI-treated mice

**Figure 4:**
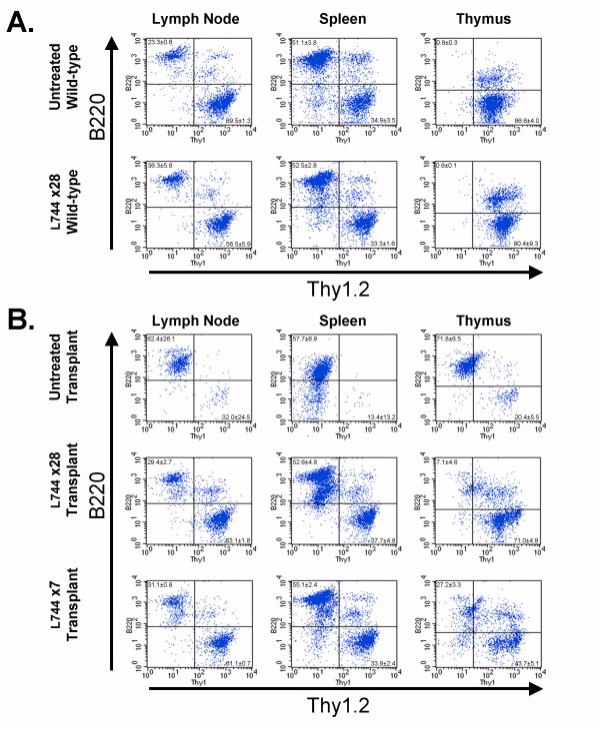
**Flow cytometry of isolated cells from mice treated with L-744,832**. Relative numbers of B- and T-cells were measured from unmanipulated C57BL/6 mice (**A**) or C57BL/6 mice that had received transgenic lymphoma transplants 28 days previously (**B**). The mice were left untreated or treated with L-744,832 for 28 days (L744 × 28) or for the final 7 days (L744 × 7). The numbers of B cells (upper left quadrant) and T cells (lower right quadrant) isolated from lymph nodes (first column), spleen (second column), or thymus (third column) were determined by flow cytometry after staining with antibodies to B220 and Thy1.2. The mean percentage and standard deviation of the B220^+ ^and the Thy1.2^+ ^cells are shown in the appropriate quadrant of each plot. There were 4 mice in all groups, except the L744 × 28 wild-type group, which had 2 mice.

Flow cytometry was used to more closely examine the effects of FTI treatment on the tumor cells and normal lymphocytes. In Figure 4A, cells from the lymph nodes, spleen, or thymus of wild-type mice were fluorescently stained with antibodies to Thy1.2 and B220 to identify T cells and B cells, respectively. Comparison of untreated mice (top panels) to mice treated for 28 days with L-744,832 (bottom panels) demonstrates that there were no significant differences in the relative numbers of B cells or T cells in the lymph nodes (untreated mice: 23.3 ± 0.6% B cells, 69.5 ± 1.3% T cells; treated mice: 36.3 ± 5.8% B cells, 56.5 ± 5.9% T cells) and spleen (untreated mice: 51.1 ± 3.8% B cells, 34.9 ± 3.5% T cells; treated mice: 52.5 ± 2.8% B cells, 33.3 ± 1.6% T cells). It did not appear that prolonged FTI treatment had substantially affected the normal distribution of lymphocytes in these organs.

Mice that received tumor transplants 28 days prior to analysis (Figure 4B, top panels) had a large increase in the number of B cells in all three lymphoid organs (lymph nodes 62.4 ± 26.1%, spleen 57.7 ± 6.9%, thymus 71.8 ± 6.5%). The presence of the tumor cells was accompanied by a large increase in the size of the affected organs (Figure 2 and data not shown). However, a small percentage of T cells remained and the total number of T cells in each organ did not decrease appreciably (Table 1). Treatment of the tumor recipient mice with L-744,832 for either 28 days (Figure 4B middle panels) or for the last 7 days (Figure 4B bottom panels) results in flow cytometry profiles that appeared much more similar to those from wild-type mice. The relative numbers of B cells and T cells in the lymph nodes (28-day treatment: 29.4 ± 2.7% B cells, 63.1 ± 1.8% T cells; 7-day treatment: 31.1 ± 0.8% B cells, 61.1 ± 0.7% T cells) and spleen (28-day treatment: 52.6 ± 4.8% B cells, 37.7 ± 4.8% T cells; 7-day treatment: 55.1 ± 2.4% B cells, 33.8 ± 2.4% T cells) returned to nearly normal. Careful comparison of the plots does reveal subtle differences, such as the presence of a significant number of B cells with a lower amount of B220 in the peripheral lymphoid organs. A small, but significant, percentage of residual B220^+^/Thy1^- ^cells remained in the thymus, even after 28 days of treatment. This appears to be due to a decrease in the number of T cells in the thymus that accompanied L-744,832 treatment (3.8 ± 1.9 × 10^7 ^T cells in untreated mice compared to 9.2 ± 0.8 × 10^5 ^T cells in tumor-recipient mice treated with L-744,832 for 28 days). The absolute number of T cells declined substantially in all of the lymphoid organs of tumor-bearing mice that were treated with L-744,832 (Table 1).

**Table 1:** Absolute number of viable B cells and T cells after L-744,832 treatment

	Lymph Nodes	Spleen	Thymus
Untreated Wild-type	B: 8.3 ± 4.4 × 10^5^	B: 3.8 ± 0.8 × 10^7^	B: 4.0 ± 3.2 × 10^5^
	T: 2.5 ± 1.2 × 10^6^	T: 2.5 ± 0.2 × 10^7^	T: 3.8 ± 1.9 × 10^7^
L744 × 28 Wild-type	B: 9.9 ± 2.8 × 10^5^	B: 1.4 ± 0.8 × 10^7^	B: 1.3 ± 0.6 × 10^5^
	T: 1.6 ± 0.9 × 10^6^	T: 9.0 ± 0.5 × 10^6^	T: 1.8 ± 0.7 × 10^7^
Untreated Transplant	B: 2.0 ± 2.5 × 10^7^	B: 1.8 ± 1.0 × 10^8^	B: 7.4 ± 5.9 × 10^7^
	T: 4.2 ± 3.9 × 10^6^	T: 4.0 ± 5.4 × 10^7^	T: 2.3 ± 2.1 × 10^7^
L744 × 28 Transplant	B: 2.3 ± 0.8 × 10^5^	B: 7.6 ± 2.9 × 10^6^	B: 9.4 ± 6.6 × 10^4^
	T: 4.8 ± 1.3 × 10^5^	T: 5.6 ± 2.4 × 10^6^	T: 9.2 ± 0.8 × 10^5^
L744 × 7 Transplant	B: 2.9 ± 0.7 × 10^5^	B: 1.0 ± 0.4 × 10^7^	B: 5.9 ± 1.9 × 10^5^
	T: 5.7 ± 1.2 × 10^5^	T: 6.5 ± 3.2 × 10^6^	T: 9.2 ± 1.9 × 10^5^

The number of viable B cells and T cells is shown for cells isolated from either the lymph nodes, spleen, or thymus of mice. The mice are either C57BL/6 (Wild-type) or C57BL/6 transplanted with tumor cells from transgenic mice 28 days earlier (Transplant). The mice were left untreated or treated with L-744,832 for 28 days (L744 × 28) or for the final 7 days (L744 × 7). Total cell number was measured with a Coulter Counter, corrected for cell viability by 7AAD staining, and then multiplied by the relative number of B or T cells determined by B220 or Thy-1 staining, respectively. Data are from the same experiment as shown in Figure 4.

### Analysis of residual tumor levels following FTI treatment

**Figure 5:**
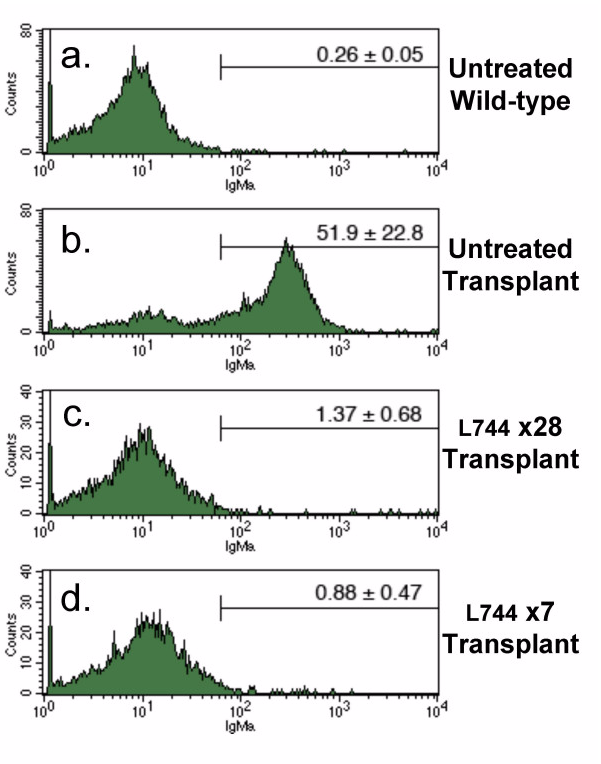
**Tumor presence in bone marrow is blocked by L-744,832**. The presence of IgM^a ^positive transgenic lymphoma cells in the bone marrow was measured using flow cytometry. C57BL/6 mice were transplanted with 10^6 ^tumor cells from an Eμ-*Myc*/BCR^HEL^/HEL mouse (panels *b*-*d*) or left unmanipulated (panel *a*). Transplant recipient mice were left untreated (panel *b*) or treated with L-744,832 for either 28 days (panel *c*) or for the final 7 days of the experiment (panel *d*). Bone marrow was harvested from the femurs and tibias of wild-type mice (panel *a*), untreated tumor recipient mice (panel *b*), 28-day treated tumor recipient mice (panel *c*) or 7-day treated tumor recipient mice (panel *d*). Isolated cells were stained for IgM^a ^and analyzed by flow cytometry. The mean percentage and standard deviation of IgM^a ^positive cells are indicated above each histogram for 4 mice in each treatment group.

The transplanted lymphoma cells accumulate in the bone marrow of recipient mice and we used this property to quantify the amount of residual tumor left after L-744,832 treatment. We used the allotypic IgM^a ^marker specific for the BCR^HEL ^transgene in donor tumor cells to distinguish them from the IgM^b^-expressing B cells normally present in C57BL/6 bone marrow. As illustrated in panel *a *of Figure 5, bone marrow from unmanipulated C57BL/6 mice did not contain IgM^a ^expressing cells. Bone marrow from mice transplanted 28 days earlier with lymphoma cells contained abundant tumor cells and an average of 51.9% of the cells were positive for IgM^a ^(Figure 5, panel *b*). The effects of L-744,832 treatment for either 28 or 7 days can be seen in Figure 5, panels *c *and *d*, respectively; the IgM^a^-expressing tumor cells in the bone marrow were significantly reduced (p < 0.01). Even after 28 days of treatment a small, but substantial number of the transgenic B cells remained when compared to mice that did not receive a transplant (p = 0.04).

**Figure 6:**
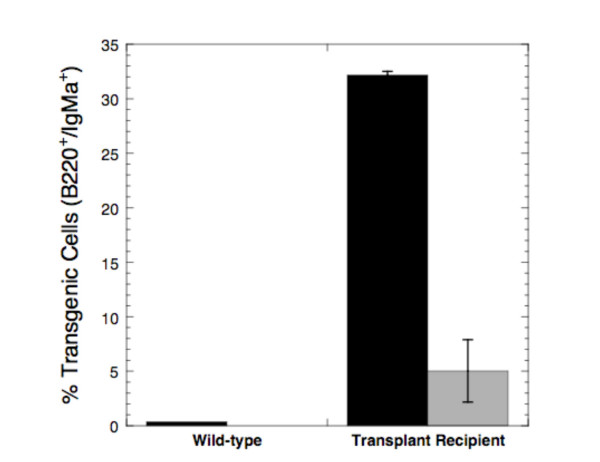
**Tumor cells decrease in SCH66336-treated mice**. Splenocytes were isolated and stained with antibodies to IgM^a ^and B220 to detect the transgenic lymphoma cells. The percent of viable splenocytes positive for both markers is shown for unmanipulated C57BL/6 mice (Wild-type) or mice transplanted with 10^6 ^tumor cells 18 days earlier (Transplant recipient). Results from untreated mice are shown with black bars and from transplant-recipient mice treated with 1.56 mg SCH66336 by oral gavage twice daily for 3 days with shaded bars. The average and standard deviation for 4 mice in each group is shown.

Similar results were seen when SCH66336 was used to treat mice transplanted with mature B cell lymphomas. When we examined isolated splenocytes for expression of IgM^a ^and B220 as markers for the lymphoma cells from the tumor transplant (Figure 6), we found that about 32% of the cells were positive for these two markers 18 days after transplantation. Treatment with SCH66336 for 3 days led to a statistically significant decrease (p < 0.05) in the number of tumor cells to 5% of splenocytes, although they were not completely eliminated.

### Sustained remissions of B cell lymphomas with brief L-744,832 treatment

**Figure 7:**
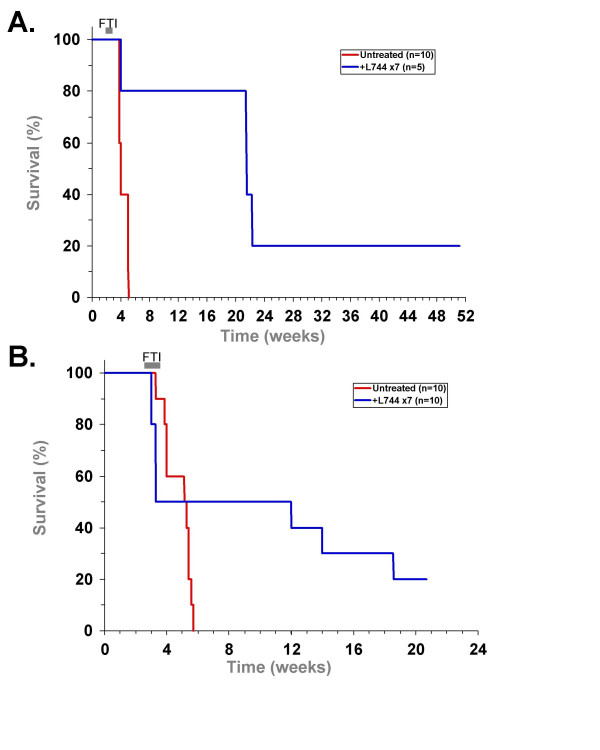
**Long-term remission and regression of lymphomas after L-744,832 treatment**. In two separate experiments (panels **A **and **B**) mice transplanted with transgenic lymphoma cells were treated for 7 days with L-744,832 starting 21 days after transplantation (blue line) or left untreated (red line). In the first experiment (panel A), 5 mice each were treated with L-744,832 or left untreated. In the second experiment (panel B), 10 mice were treated with L-744,832 and 5 mice were untreated. Mice were then monitored for signs of lymphadenopathy and euthanized when moribund.

We next examined whether the treatment of tumor-bearing mice for 7 days with L-744,832 could cause long-lasting remissions from acute B cell lymphoma. Two separate experiments were conducted with either 5 mice (Figure 7A) or 10 mice (Figure 7B) that had received 10^6 ^tumor cells and were then treated with L-744,832 for 7 days, starting 21 days after the transplantation, when lymphomas were first evident by external examination in all transplant recipient mice. In the first experiment, the untreated mice became moribund and were euthanized between 5 and 6 weeks after transplantation. One of the treated mice did not appear to respond to L-744,832 treatment and tumor progression was similar to untreated mice. Three of the remaining 4 treated mice showed temporary remissions of lymphoma. There were no signs of lymphadenopathy in these mice until approximately 6 weeks after the treatment ended. These 3 mice survived for approximately 17 weeks and developed very large, indolent lymphomas that were not as aggressive as the original transplanted cells, which would be expected to cause morbidity within 3 weeks of visible lymphadenopathy. Lymphadenopathy never returned in the fifth treated mouse and it was euthanized after 52 weeks. Necropsy showed no signs of splenomegaly or lymphadenopathy (data not shown).

In the second experiment (Figure 7B), the untreated tumor recipients showed visible signs of tumor at 3 weeks and had to be euthanized between 3 and 6 weeks. Ten other tumor recipient mice were treated with L-744,832 starting 21 days after transplantation and 5 of these mice died during treatment, apparently as a result of the treatment. FTI treatment in these 5 mice caused their condition to worsen rapidly and death typically occurred within an hour of the first or second drug administration. Similar acute reactions to FTI treatment were observed in other mice with large tumors (data not shown). These adverse reactions were not as severe in mice that did not receive tumor transplants or in mice that were treated before large tumors were present. This observation is consistent with L-744,832 treatment causing tumor lysis syndrome in mice with a large tumor burden, similar to what is seen in human Burkitt's lymphoma patients treated with current chemotherapy treatment [36]. Tumor lysis syndrome is typically managed effectively in at-risk B cell lymphoma patients [37]. Of the remaining 5 mice, 3 had temporary remissions and later developed large lymphomas, becoming moribund between 8 and 15 weeks after treatment. The lymphadenopathy did not return in the remaining 2 treated mice and they remained healthy 26 weeks after treatment when they were euthanized, showing no evidence of splenomegaly or lymphadenopathy. From these two experiments, combined, we found that 40% (6/15) of the mice died as a possible effect of L-744,832 treatment, and of the mice that survived initial treatment, 33% (3/9) showed long term recovery with no evidence of lymphoma.

## Discussion

### Selectivity of FTIs for tumor cells

In the mouse lymphoma model used here, two signaling pathways have been manipulated to generate tumor B cells that recognize a self antigen. Continuous antigen receptor stimulation, provided by the co-expression of BCR^HEL ^and HEL, drives B cell proliferation. This would normally lead to cell death or B cell anergy [38]; however, the over-expression of *c-Myc *in these autoreactive B cells overcomes tolerance, allowing the tumor cells to survive and hyperproliferate [29]. The cooperation of *c-Myc *over-expression and antigen receptor signaling in tumorigenesis may be related to the observation that the Myc transcription factor is required for cytokine-dependent survival signaling in lymphocytes [29]. Over-expression of *c-Myc *in transgenic B cells renders them independent of cytokines for proliferation and survival *in vitro *[29]. We reasoned that one or more farnesylated proteins were likely to be critical for the antigen receptor or cytokine receptor/Myc signaling pathways. Therefore, inhibition of farnesyl transferase might restore normal regulation to the dysplastic B cells. Our results clearly support this hypothesis and strongly implicate at least one farnesylated protein in either of these two signaling pathways. This result, alone, is hardly surprising, but what we find most interesting is the apparent specificity of the FTI for the tumor B cells compared to nontransformed lymphocytes. When the proliferation of activated B cells and the transformed B cells were compared *in vitro*, the tumor cells were approximately 10-fold more sensitive to L-744,832 treatment than naïve B lymphocytes stimulated with antigen receptor and CD40 antibodies (Figure 1).

Similar selectivity for tumor cells was observed *in vivo*. FTI treatment of mice for as little as three days eliminated > 90% of the tumor cells, while only slightly affecting the normal lymphocyte populations in the same mice (Figures 2, 3, and 4). When mice without tumors were treated with L-744,832 for as long as 28 days, no significant changes were seen in the development of cells in the lymphoid lineages (Figure 4), a process that involves antigen receptor signaling during positive and negative selection of immature lymphocytes. This suggests that the tumor B cells are more susceptible to loss of the functional targets of FTIs than normal B cells. There are at least two explanations for the greater sensitivity of the transformed cells. FTIs could suppress lymphocyte activation specifically under conditions of very high antigenic stimulation, as in the case of a self-antigen, or the breach of tolerance by *c-Myc *over-expression could render the B cells sensitive to the FTI. These possibilities have implications for the possible use of FTIs to treat autoimmune disease or in conditions of alloreactivity.

Further investigation will be necessary to identify the mechanism that FTIs may be affecting in order to exert the biological effects that we observe. Farnesyl transferase is estimated to modify 40 to 50 different proteins in mammalian cells and the critical anti-neoplastic targets of FTI treatment remain unknown [1,39]. Proteins whose inhibition can mediate anti-cancer effects of FTIs include RhoB [40] and the centromeric proteins CENP-E and CENP-F [41]; most likely a combinatorial effect on many farnesylated proteins is necessary for the cytostatic and cytotoxic effects of FTIs [42].

The demonstration that several different FTIs can block the production of T_H_1 and T_H_2 cytokines from T cells in culture [43] may provide a partial explanation for the effects of these drugs in our experiments. Marks *et al*. demonstrate that FTI treatment of activated T cell clones blocked the secretion of IL-2, interferon-γ, IL-4, and IL-5. Cytokine signaling plays a critical role in lymphocyte proliferation and survival, as well as establishing tolerance to self antigens [44]. Therefore, it is possible that FTI treatment affects the hyperproliferation of the self-reactive B cells by interfering with cytokine production. However, the overexpression of *Myc *in the self-reactive B cells studied here presumably substitutes for IL-4 receptor activation and the transgenic B cells do not require IL-4 production for survival and proliferation [43]. Therefore, we view it as unlikely that the *Myc*-overexpressing B cells would be affected by an FTI-dependent block of IL-4 production. However, FTI treatment may decrease the production of cytokines by the transformed B cells, which may, in turn, affect the regulatory environment surrounding the B cells and help to restore self tolerance to the HEL self antigen.

### Comparison to other FTI preclinical studies

FTI treatment has been shown to have anti-tumor activity in several other mouse cancer models. The mature B cell lymphoma studied here is similar to the non-Hodgkin lymphoma studied by R. Mangues, *et al*., who demonstrated that L-744,832 could prevent the formation of diffuse large B cell splenic lymphomas in mice that expressed an activated *N-Ras *oncogene under the MMTV promoter [8]. L-744,832 has also been shown to cause regression of mammary and salivary tumors in mice expressing MMTV- *v-H-Ras *alone [45], or together with MMTV- c-*Myc *[7]. Unlike the above transgenic tumor models, the murine lymphoma studied here does not contain an activated *Ras *transgene but may express a spontaneously activated *Ras *allele. Such mutations are not commonly found in B cell non-Hodgkin lymphomas in humans [46], however, we cannot rule out that a spontaneous mutation occurred at one of the *Ras *alleles during the process of tumorigenesis in our mouse model. FTIs have mixed results with other mouse tumors that are not known to harbor *Ras *mutations. For example, the mammary tumors in MMTV- *c-Neu *mice do not respond to L-744,832 [7], but the pre-B-cell leukemia in *BCR/Abl *transgenic mice regressed upon treatment with SCH66336 [21]. Our results are consistent with other models that do not use *Ras *mutations as an initiating tumorigenic event but have shown that FTI treatment can still effectively block tumor growth.

### Implications for treatment of human B cell lymphomas

Our preclinical results with this mouse lymphoma model indicate that patients with mature B cell lymphomas should be considered for inclusion in FTI clinical trials. One of the important features of this mouse model is the self-reactivity of the transformed B cells. Although FTIs do not appear to block proliferation of nontransformed B cells in response to antigen receptor stimulation, these drugs were able to block proliferation and induce cell death in the self-reactive transformed B cells. Antigen receptor activation by self or environmental antigens may contribute the generation of certain B cell malignancies in humans [35]. Recognition of autoantigens or foreign antigens from a chronic infection appears to be an important feature of a substantial fraction of B cell chronic lymphocytic leukemias [47,48], follicular lymphomas [49], and certain other leukemias and lymphomas [35]. We suggest that FTIs may be able to block the proliferation of BCR-expressing B cell lymphomas by interfering with the antigen receptor and/or cytokine signaling pathways within the transformed cells.

The lymphoma in the mice used in this study most closely resembles Burkitt's lymphoma, and we propose that FTIs might be a useful addition to the chemotherapy for this cancer. The Raji Burkitt's lymphoma cell line has been shown to be sensitive to a geranylgeranyl transferase inhibitor and to lovastatin but less sensitive to an FTI [50]. Together with our results, this suggests that both FTIs and geranylgeranyl transferase inhibitors should be investigated as possible Burkitt's lymphoma treatments. Initial clinical trials using FTIs focused on cancers that were likely to harbor activating Ras mutations, such as pancreatic cancer. However, subsequent experience has led to the inclusion of many additional patient groups in FTI clinical trials. For example, SCH66336 is undergoing a phase III clinical trial on myelodysplastic syndrome and chronic myelomonocytic leukemia (Study ID P02978). However, mature B cell lymphomas, such as Burkitt's, are not specifically targeted in any of the current FTI clinical trials, perhaps because of the high success rate of current therapies for Burkitt's lymphoma.

Treatment of Burkitt's lymphoma typically involves a combination of chemotherapies: cyclophosphamide, etoposide, vincristine, bleomycin, doxorubicin, methotrexate, and prednisone [36,51]. Recent evidence has emerged that the anti-CD20 antibody Rituximab may increase the efficacy of treatment in Burkitt's lymphoma, as it does for other mature B cell lymphomas that express CD20 [52]. Although the aggressive combination therapy leads to long-term remission in most patients, at least 20% will relapse within five years and many patients are not able to complete this regimen due to severe side effects. In addition, success rates are considerably lower in older adults and in developing countries where Burkitt's lymphoma is most common [36]. The addition of an FTI to this treatment, possibly in combination with Rituximab, could increase the success rate, allow comparable success with less toxic chemotherapies, or allow more effective treatment of relapsing or refractory cases. Importantly, in regions of the world where Burkitt's lymphoma is most common, conventional treatments are less successful and an orally effective FTI could, in combination with other treatment, have a positive effect on the outcome of this disease.

## Conclusion

Using an Eμ-*Myc*/BCR^HEL^/HEL transgenic mouse model of a mature B cell lymphoma, we found that FTI treatment could block the hyperproliferation and survival of this lymphoma *in vitro *and *in vivo*. Specifically, we showed that proliferation in culture of the transformed B cells from the transgenic mice was more sensitive to L-744,832 treatment than nontransformed activated B cells. In mice we showed that either L-744,832 or SCH66336 treatment caused a rapid loss of tumor cells transplanted from a transgenic mouse. Treatment with L-744,832 for seven days resulted in long term remissions from disease in one third of mice that survived treatment. These results suggest that FTIs should be considered as adjuncts for combination therapies or treatments for refractory cases in patients with mature B cell lymphomas, including Burkitt's lymphoma and other lymphomas that may be dependent on antigen receptor activation. It would be particularly interesting to see if FTI treatment would be effective when combined with Rituximab, an anti-CD20 antibody that can be very effective at treating certain B cell lymphomas.

## Methods

### Mice

Eμ-*Myc*/BCR^HEL^/HEL transgenic mice have been previously described [29]. Eμ-*Myc *transgenic mice that overexpress *c-Myc *in B cells after the Pre/Pro-B cell stage of development [53] were obtained from The Jackson Laboratory (Bar Harbor, ME). BCR^HEL ^transgenic mice that expressed a pre-rearranged, HEL-specific B cell receptor from the endogenous Ig promoter [38] were kindly provided by Jason Cyster (University of California, San Francisco). The HEL transgenic mice that expressed secreted HEL from a metallothionine promoter [38] were also provided by Jason Cyster. Mice were maintained on a C57BL/6 background and genotyped by PCR as previously described [38,53,54]. Eμ-*Myc*/BCR^HEL^/HEL transgenic mice were obtained by crossing Eμ-*Myc *mice with mice heterozygous for both BCR^HEL ^and HEL transgenes. All mice were maintained humanely according to protocols approved by the Bucknell University Institutional Animal Care and Use Committee or the UCSF Committee on Animal Research.

Lymphomas were transplanted by removing the spleen and three pairs of lymph nodes (inguinal, axillary, and brachial) from an Eμ-*Myc*/BCR^HEL^/HEL transgenic mouse with externally evident lymphoma. Single cell suspensions were prepared separately from spleens and the lymph nodes by macerating organs through a 60 μm mesh screen (Sigma, St. Louis, MO). Red blood cells were lysed using 17 mM Tris, pH 7.65, 135 mM NH_4_Cl buffer and the remaining splenocytes and lymphocytes were resuspended in complete RPMI media (RPMI 1640 with L-glutamine, penicillin/streptomycin, non-essential amino acids, 2 mM HEPES, 2 mM sodium pyruvate, 10 mM β-mercaptoethanol (all from Invitrogen, Grand Island, NY), and 10% heat-inactivated fetal bovine serum (HyClone, Logan, UT)). Cells were counted using a hemocytometer and Trypan blue staining (Sigma). Cells were washed three times with Hank's Buffered Salt Solution (HBSS, Invitrogen) and each C57BL/6 recipient mouse was intravenously injected with 5 × 10^5 ^each of cells from the lymph node and spleen.

### Farnesyl Transferase Inhibitors

L-744,832 (BIOMOL, Plymouth Meeting, PA) was dissolved in HBSS at 2.5 mg/ml and filtered through a 0.2 μm syringe filter for sterilization. Aliquots were frozen at -20°C and used within one month. Mice were treated with L-744,832 by tail vein injection of 0.25 ml once daily. SCH66336 was generously provided by Robert Bishop (Schering Plough Research Institute, Kenilworth, NJ). SCH66336 was dissolved in 20% (w/v) hydroxypropyl-β-cyclodextrin (Sigma) in HBSS at 6.25 mg/ml and filtered through a 0.2 μm syringe filter [19]. Mice were treated with SCH66336 by oral gavage with 0.25 ml every 10 to 14 hours. SCH66336 aliquots were frozen and stored at -20°C.

### Proliferation Assays

Cell proliferation was measured in culture by labeling with the dye 5-(6)-carboxyfluorescein diacetate, succinimidyl ester (CFSE; Invitrogen). Splenocytes were isolated from either a BCR^HEL ^transgenic mouse or a moribund Eμ-*Myc*/BCR^HEL^/HEL transgenic mouse as described above. T cells were depleted using anti-Thy 1.2 paramagnetic beads as described by the manufacturer (Invitrogen). Cells were resuspended at 5 × 10^6 ^cells/ml in HBSS and labeled for 5 minutes at room temperature by the addition of an equal volume of 1.0 μM CFSE. The labeling reaction was quenched by the addition of 2 volumes of fetal bovine serum, further diluted with complete RPMI, and cells were then washed three times with complete RPMI. Cells were then placed in culture at 37°C in 5% CO_2 _at 5 × 10^5 ^cells/ml in complete RPMI with 2 μg/ml goat anti- mouse IgM, μ-specific (Jackson Immunoresearch, West Grove, PA), 1 μg/ml anti-mouse CD40 (BD Biosciences, San Diego, CA), and L-744,832, as indicated. A Becton Dickinson FACScan flow cytometer was used to measure proliferation after 3 days. Viable cells were gated based on forward and side scatter. Between 2500 and 10,000 viable cells were analyzed for each sample by measuring the CFSE fluorescence associated with each cell.

### Flow Cytometry

Single cell suspensions from the lymph nodes, spleen, or thymus of each mouse were prepared as described above. Cells were resuspended in FACS Buffer and incubated with anti-CD16/CD32 (BD Biosciences) to block Fc receptors. Cells were then labeled, as indicated, with fluorescent antibodies to B220, Thy 1.2, or IgM^a ^(BD Biosciences) diluted 1:50 with FACS Buffer. Unbound antibody was removed by washing with FACS Buffer and cells were fixed with 1% paraformaldehyde (Sigma) in HBSS. Viable cells were gated based on forward and side scatter. For measurement of absolute cell numbers, a Coulter Counter (Beckman Coulter, Fullerton, CA) was used to measure cell density and, separately, a sample was labelled with 7-aminoactinomycin D (7AAD; Invitrogen) and analyzed by flow cytometry as recommended by the manufacturer to determine cell viability.

## Competing interests

Schering-Plough Research Institute provided SCH66336 for use in these studies.

## Authors' contributions

KAF participated in acquiring, analyzing, and interpreting the data. YR participated in acquiring and analyzing the data involving L-744,832 and conceived of the Eμ-*Myc*/BCR^HEL^/HEL transgenic mouse model. SC participated in acquiring and analyzing the data involving SCH66336. KAF, YR, and JMB were involved in the conceptual design and provided important intellectual contributions. KAF wrote the manuscript with important revisions provided by JMB. All authors read and approved the final manuscript.
